# CT-based age-related trabecular redistribution in the proximal femur and its association with fracture patterns in elderly individuals with and without diabetes

**DOI:** 10.3389/fendo.2026.1830903

**Published:** 2026-09-29

**Authors:** Shuo Chen, Xuefeng Ma, Qian Meng, Yanhong Dong, Yingying Cheng, Lei Gao, Jie Tian, Dandan Yang, Ao Qi, Ping Zhang

**Affiliations:** 1 Radiology Department, The 3rd Hospital of Hebei Medical University, Shijiazhuang, China; 2 Radiology Department, Hebei Chest Hospital, Shijiazhuang, China; 3 Radiology Department, Beijing Geriatric Hospital, Beijing, China

**Keywords:** age, CT, diabetes, osteoporosis, proximal femoral fracture, trabecular

## Abstract

Proximal femoral fractures are the most common fall-related fractures in the elderly. In our clinical work, we observed a phenomenon of trabecular redistribution in the proximal femur of older individuals. To investigate the distribution of bone trabeculae (compression and tension trabeculae) in the proximal femur, their changes with age, and their impact on fracture risk, we retrospectively analyzed hip computed tomography (CT) images and measured CT values in six different regions of the proximal femur: the primary compression trabecula, overlapping area, primary tension trabecula, secondary compression trabecula, the greater trochanter and lateral of primary tension trabecula. With increasing age, the CT values in six regions decreased (p<0.01). The ratio of the primary compression trabecular area to the overlapping area remained relatively stable (F = 1.84, p=0.12), whereas the ratio of the primary tension trabecular area to the overlapping area decreased (F = 9.67, p<0.01). Elderly patients with proximal femoral fractures exhibited significant trabecular redistribution. Notably, compared to non-diabetic elderly patients with proximal femoral fractures, those with diabetes and femoral fractures showed a marked decrease in the ratio of the primary compression trabecular area to the overlapping area relative to normal controls (d=-0.40, p<0.05). These findings may improve understanding of altered fracture susceptibility in elderly diabetic patients. In conclusion, although bone density in the proximal femur generally declines with age, trabecular redistribution occurs, characterized by a relative reduction in tension trabeculae and a relatively stable ratio of compression trabeculae. However, in elderly diabetic patients with fractures, the compression trabeculae fail to maintain this stable ratio, which may reflect altered trabecular adaptation associated with fracture susceptibility.

## Introduction

As population aging intensifies, osteoporosis and its associated fractures have become increasingly prominent. Osteoporosis is a major cause of fractures in the elderly. Osteoporosis is characterized primarily by the loss of trabecular rather than cortical bone, a feature considered central to increased fracture susceptibility (1). The Singh index, introduced in 1970, semi-quantitatively classifies osteoporosis-related visual changes in the tensile and compressive trabeculae of the proximal femur into six grades, ranging from trabecular thickening to complete disappearance, with lower grades indicating more severe osteoporosis (2).Hip fractures are the most common type of osteoporotic fracture, and approximately 20% of patients with hip fractures die prematurely within one year (3, 4). Fragile hip fractures represent the most severe type of osteoporotic fracture due to their high disability and mortality rates. It is predicted that by 2050, the global number of hip fractures will increase from 1.6 million in 2000 to 4.5 million, with Asia accounting for 50% of cases, particularly in China (5–7).

Osteoporosis leads to a reduction in the number and structural integrity of trabeculae, thereby affecting bone mechanical properties and stability. With advancing age, skeletal system degeneration results in decreased bone density and morphological changes in trabeculae, which gradually become thinner, sparser, and may even break or disappear. The number, quality, and distribution of trabeculae play important roles in the onset and progression of osteoporosis. In our daily practice, we observed a phenomenon of trabecular redistribution in the proximal femur of elderly individuals. A review of the literature revealed no relevant studies on age-related factors affecting the distribution of compression and tension trabeculae. Investigating the relationship between trabecular strength, density, and morphology can help explain measured bone strength. Related biomechanical studies have also noted that a lack of tensile strength data precludes definitive conclusions on ultimate strength-density-morphology relationships (8). Studying age-related changes in tension and compression trabeculae will enhance our understanding of osteoporotic fractures.

Increased fracture risk is an emerging and severe complication of diabetes. In type 1 diabetes, bone mass and bone strength are reduced, leading to up to a fivefold increase in fracture risk throughout life. In type 2 diabetes, fracture risk is increased despite normal bone mass (9). Some patients with type 2 diabetes exhibit higher bone density but poor bone quality. What differences exist in trabecular bone arrangement between diabetic patients and those with age-related osteoporosis? Do these differences contribute to the increased fracture risk in diabetic patients? These questions form the focus of our study.

## Materials and methods

### Participant selection and study design

#### Exclusion criteria

Individuals with BMI <18.5 kg/m² or BMI ≥30 kg/m² were excluded. Patients with chronic kidney disease (stage 4–5), hyperparathyroidism, hyperthyroidism, rheumatoid arthritis, or chronic liver disease were excluded.

Retrospective analysis of hip joint CT images of 531 patients aged 21 to 70 from October 2023 to July 2024. All CT images were collected by 128-slice CT (Edge, Siemens healthcare). The scanning parameters were as following: tube voltage 120 kV, tube current 130mAs, slice thickness 1.0 mm, matrix 512×512, FOV215×215mm2. All CT scanners were calibrated monthly using the ESP_145 European body phantom. Patients with metabolic diseases or tumors that may affect bone metabolism—including secondary osteoporosis, hyperparathyroidism, chronic steroid use, and renal osteodystrophy, among others—were excluded from the study. For participants with bilateral hip imaging, one side (left or right) was randomly selected using a random number table; for those with unilateral imaging, the available side was directly used for analysis. To analyze the relationship between age and trabecular bone distribution, patients were divided into five groups: 21-30, 31-40, 41-50, 51-60, and 61–70 years.

We also collected the CT examination data from patients aged 61–70 years with proximal femoral fractures (n=203) from 2022 to 2024. These were divided into diabetic (n=94) and non-diabetic (n=109) groups. All diabetic patients included in this study had type 2 diabetes mellitus (T2DM), diagnosed according to the American Diabetes Association (ADA) criteria (fasting plasma glucose ≥ 7.0 mmol/L, 2-hour postload glucose ≥ 11.1 mmol/L during oral glucose tolerance test, or HbA1c ≥ 6.5%), or a documented history of T2DM with ongoing glucose-lowering therapy. CT values of proximal femur in 6 regions were measured on the healthy side to analyze the effect of trabecular redistribution on fracture and the impact of diabetes on trabecular redistribution and fracture (**Figure 1**).

**Figure 1:**
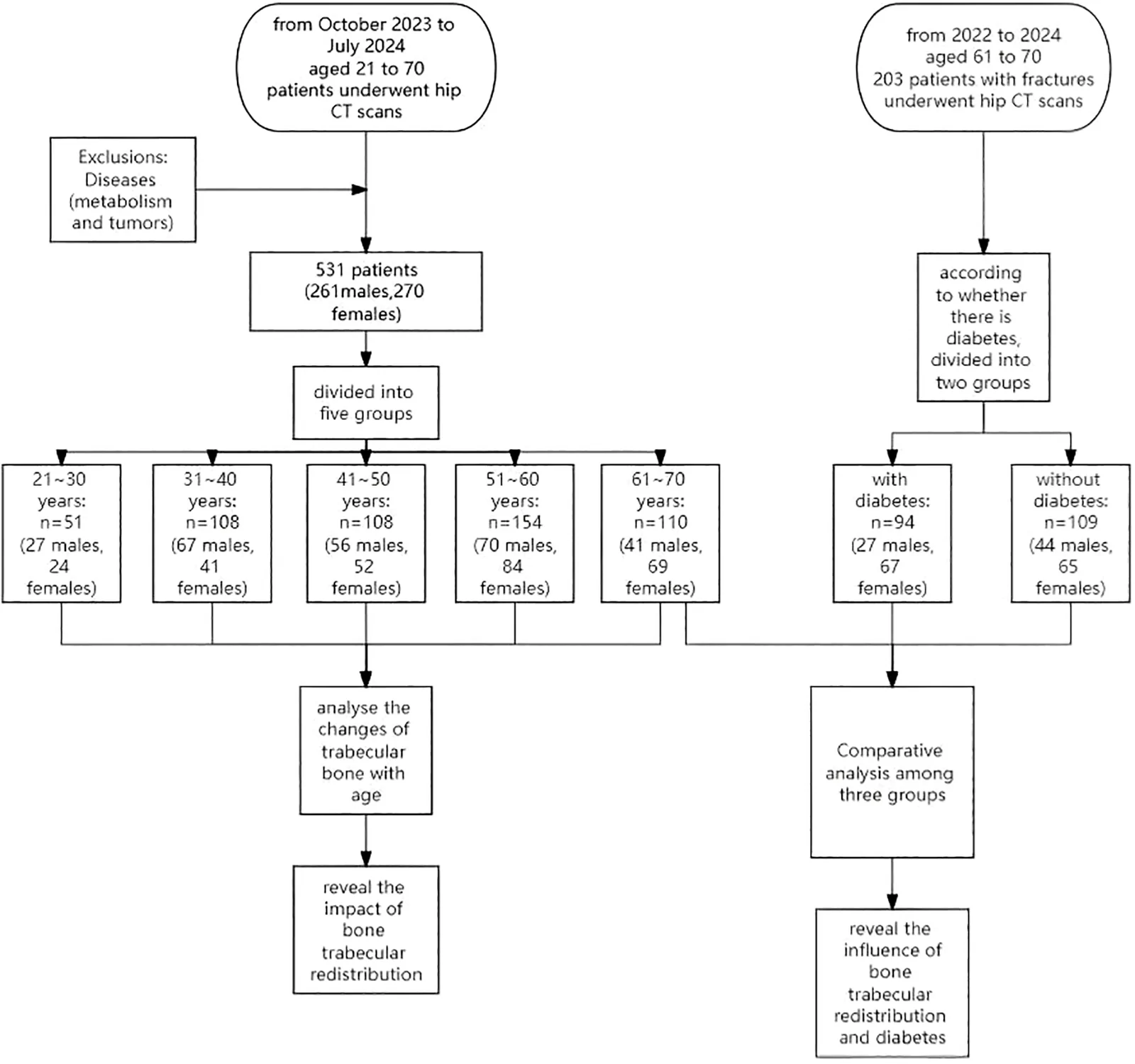
Patient selection flowchart. CT, Computed Tomography.

### CT image analysis

CT values (HU) were measured using a consistent window level and width (window level 450 HU, window width 1500 HU) for all images. CT values of the proximal femur were measured in six predefined regions (**Figure 2**): the primary compression trabecula, overlapping area, primary tension trabecula, secondary compression trabecula, greater trochanter, and lateral aspect of the primary tension trabecula. Using CT post-processing software, circular regions of interest (ROIs) with an area of approximately 0.3 square millimeters were manually placed in each region, carefully avoiding cortical bone to minimize partial volume effects while preserving regional anatomical specificity. The anatomical landmarks for each region were defined as follows: (1) primary compression trabecula – the center of the femoral head–neck junction; (2) overlapping area – the intersection of the primary compression and tension trabeculae, approximately 1 cm medial to the greater trochanter; (3) primary tension trabecula – the lateral femoral neck along the direction of the tension trabeculae; (4) secondary compression trabecula – the inferomedial femoral neck near the calcar femorale; (5) greater trochanter – the center of its apophysis; and (6) lateral aspect of the primary tension trabecula – the lateral cortex distal to the gluteus minimus insertion. Ratio 1 was defined as the ratio of CT values in the primary compression trabecular region to those in the overlapping region, and Ratio 2 was defined as the ratio of CT values in the primary tension trabecular region to those in the overlapping region.

**Figure 2:**
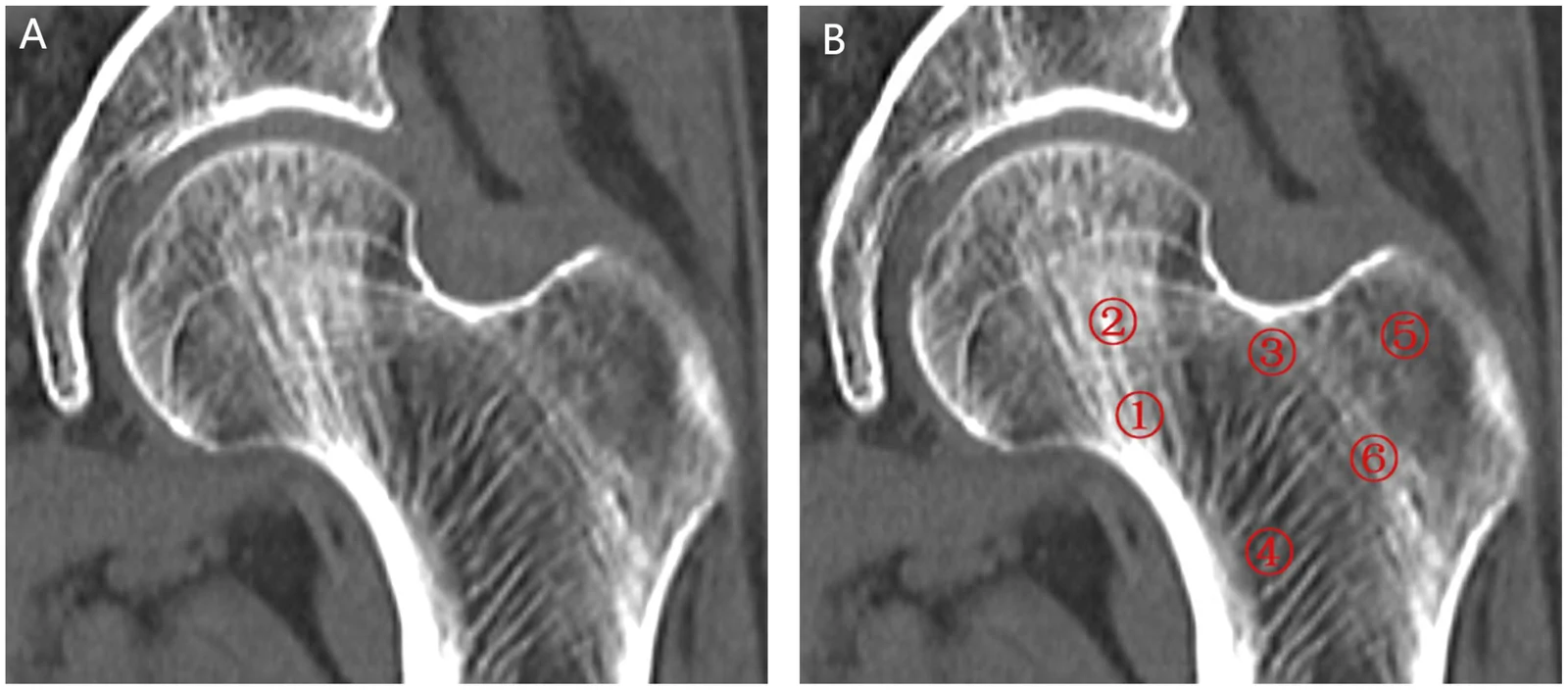
**(A)** Original CT image **(B)** The annotated CT image displays six measurement areas of the proximal femur. ①=primary compression trabecula, ②=overlapping area, ③=primary tension trabecula, ④=secondary compression trabecula, ⑤=the greater trochanter, ⑥=lateral of primary tension trabecula.

To ensure consistent slice selection across all 531 patients, all CT volumes were reformatted to a true coronal plane aligned with the femoral shaft axis and the center of the femoral head. The target slice was defined as the single image that simultaneously displayed: (a) the fovea capitis femoris, (b) the maximum mediolateral diameter of the femoral neck, and (c) the craniocaudal midpoint of the lesser trochanter. Slice selection was performed independently by two trained observers. In cases of disagreement (defined as a slice difference of ≥2 mm), a third senior observer made the final decision. Twenty randomly selected cases were re-measured by two observers (blinded) and by the same observer after two weeks.

### Statistical analysis

Statistical analyses were performed using SPSS version 29.0 (IBM) and GraphPad Prism 10.4, in consultation with a biostatistician. After normality testing, if the data meet normal distribution and homogeneity of variances, one-way ANOVA is used, otherwise the Kruskal-Wallis H test is applied. Normality was assessed using the Shapiro-Wilk test, and homogeneity of variances was evaluated using Levene’s test.Baseline characteristics were presented as means and standard deviations (SDs) for continuous variables. One-way analysis of variance (ANOVA) was used to assess the relationship between age and trabecular bone distribution. The effects of trabecular redistribution on fractures and the effects of diabetes on trabecular redistribution and fractures were investigated by one-way ANOVA and Bonferroni-corrected *post hoc* multiple comparisons. Two-way analysis of variance (ANOVA) was used to examine the interaction of age or diabetes with sex. The effect of fracture site on trabecular redistribution was investigated using one-way analysis of variance (ANOVA) with *post-hoc* multiple comparisons adjusted by the Bonferroni correction. P < 0.05 was considered statistically significant.

## Results

### Reproducibility

Twenty randomly selected cases were re-measured by two observers (blinded) and by the same observer after two weeks. The intraclass correlation coefficient (ICC) was calculated, and results have been added to the Results section and a new supplementary table. Interobserver ICC ranged from 0.82 to 0.94, and intraobserver ICC from 0.80 to 0.94, indicating good to excellent reliability. See **Supplementary Tables 1** and **2** for details.

### Effects of age and gender on trabecular distribution

The relationship between age and trabecular distribution is shown in **Table 1** and **Figure 3**. The CT values of all regions decreased with age (P<0.01). Ratio 1 remained relatively stable (F = 1.84, p=0.12), whereas ratio 2 decreased (F = 9.67, p<0.01). As shown in the **Figure 4**, there were sex differences in trabecular changes. Primary compression trabecular CT values remained relatively stable with age in men but showed a declining trend in women. Ratio1 remained stable in women, whereas ratio1 in elderly men was higher than that in middle-aged and younger men.

**Table 1:** One way ANOVA of age group and trabecular bone distribution.

Area	Age group (mean ± standard deviation)					*F*	*p*
	21~30 (*n* = 51)	31~40 (*n* = 108)	41~50 (*n* = 108)	51~60 (*n* = 154)	61~70 (*n* = 110)		
1	382.13 ± 96.99	353.14 ± 95.83	368.14 ± 116.03	338.31 ± 105.64	304.71 ± 120.03	6.833	0.000**
2	316.39 ± 93.35	298.90 ± 98.99	300.56 ± 99.85	274.64 ± 88.52	243.21 ± 101.00	8.173	0.000**
3	236.22 ± 82.18	163.43 ± 59.38	163.85 ± 70.29	141.80 ± 65.88	109.16 ± 54.46	35.861	0.000**
4	192.71 ± 78.41	148.23 ± 68.73	159.81 ± 72.64	127.55 ± 67.89	101.49 ± 56.07	20.500	0.000**
5	84.93 ± 46.56	62.88 ± 34.10	65.22 ± 40.36	63.25 ± 44.81	39.72 ± 44.84	11.564	0.000**
6	125.62 ± 63.68	92.63 ± 46.02	99.53 ± 53.42	83.94 ± 53.95	53.45 ± 51.83	19.784	0.000**
Ratio1	1.24 ± 0.27	1.24 ± 0.30	1.26 ± 0.29	1.27 ± 0.27	1.35 ± 0.49	1.835	0.121
Ratio2	0.77 ± 0.25	0.59 ± 0.27	0.58 ± 0.26	0.55 ± 0.27	0.49 ± 0.28	9.667	0.000**

*p < 0.05, **p < 0.01 1=primary compression trabecula, 2=overlapping area, 3=primary tension trabecula, 4=secondary compression trabecula, 5=the greater trochanter, 6=lateral of primary tension trabecula.

**Figure 3:**
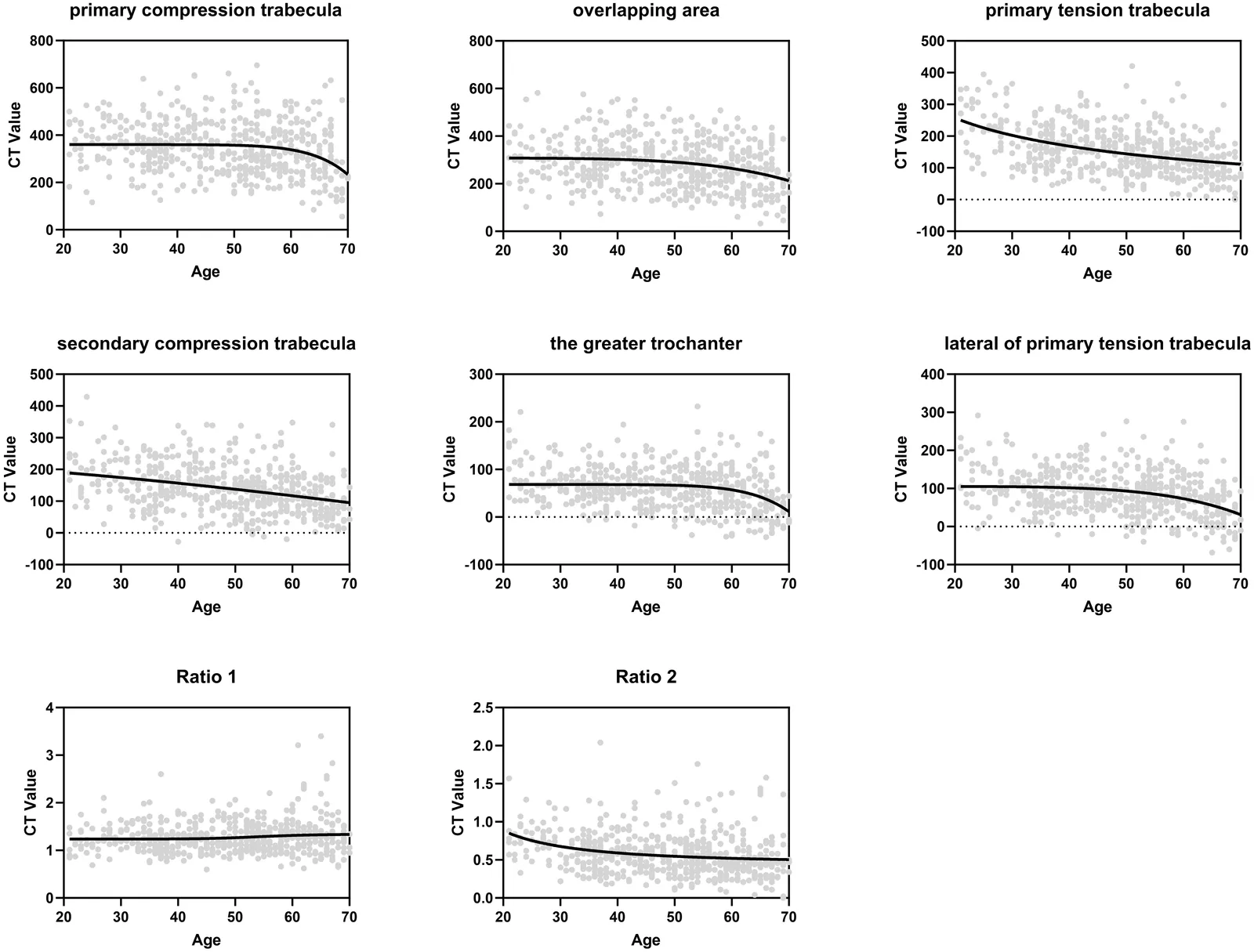
Changes in CT values of bone trabeculae in the measurement areas with age.

**Figure 4:**
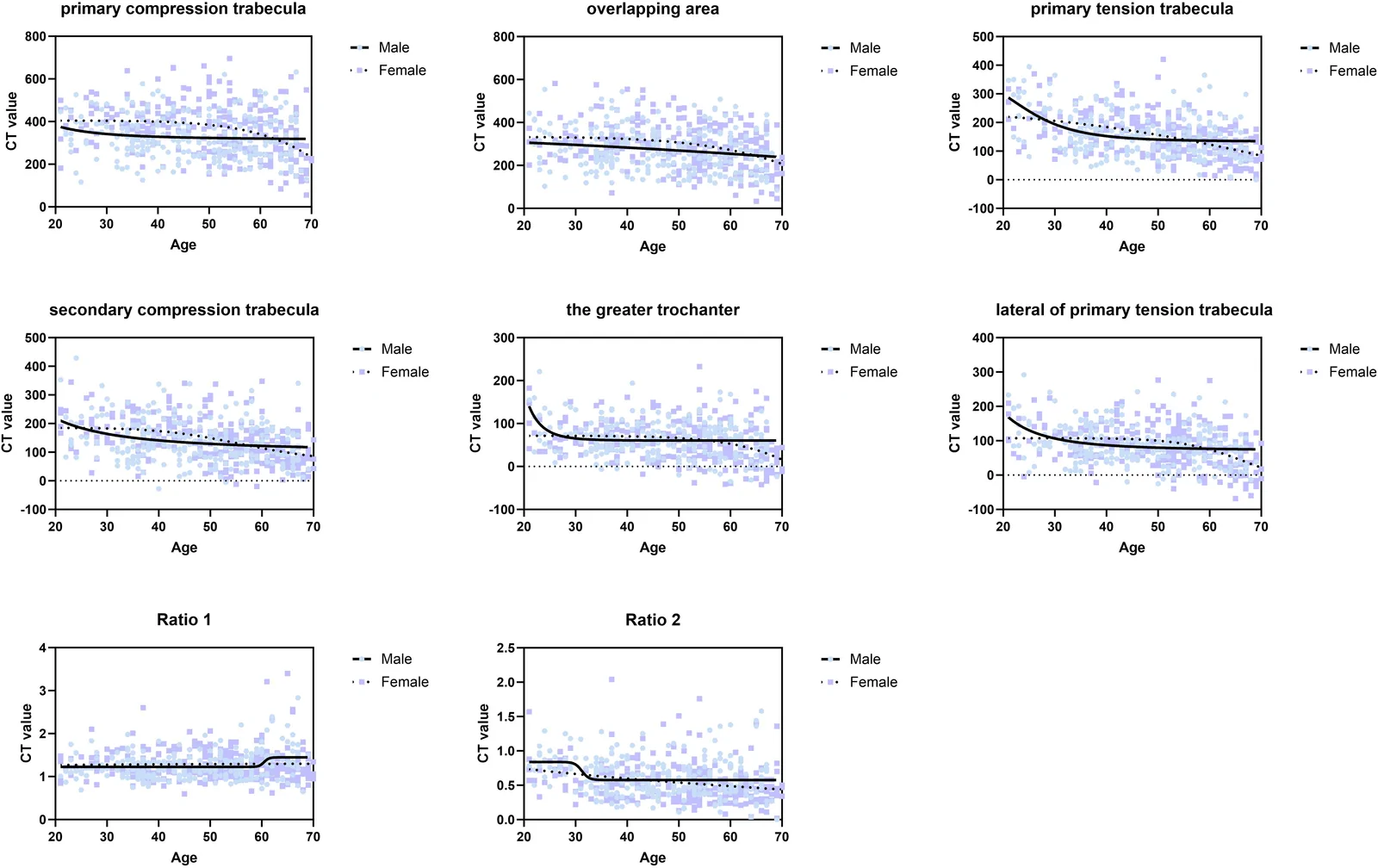
Changes in CT values of bone trabeculae in the measurement areas classified by gender with age.

For Ratio1, under the conditions of α = 0.05 and 80% power, the minimum detectable effect sizefor the current five-group sample size (n = 531) was Cohen’s f = 0.150 (a small effect). The observed effect size in the current experimental design was f = 0.118, providing 62% power to detect a true between-group difference, which is below the 80% benchmark.

### Effect of the trabecular redistribution on fracture and impact of diabetes

The effects of trabecular redistribution on fractures and the impact of diabetes are shown in **Tables 2**, **3**.

**Table 2:** One way ANOVA of 61–70 years old normal group and patients with and without diabetic fracture.

Area	Patient type (mean ± standard deviation)			*F*	*p*
	Fracture with diabetes (*n* = 94)	Fracture without diabetes (*n* = 109)	Normal (*n* = 110)		
1	360.61 ± 81.90	369.78 ± 106.50	304.71 ± 120.03	12.157	0.000**
2	308.92 ± 68.19	292.87 ± 71.63	243.21 ± 101.00	18.138	0.000**
3	93.57 ± 51.86	86.35 ± 52.66	109.16 ± 54.46	5.269	0.006**
4	77.51 ± 48.88	79.25 ± 62.87	101.49 ± 56.07	5.941	0.003**
5	35.53 ± 42.28	26.73 ± 38.77	39.72 ± 44.84	2.710	0.068
6	60.72 ± 45.42	51.83 ± 50.08	53.45 ± 51.83	0.909	0.404
Ratio1	1.19 ± 0.23	1.30 ± 0.42	1.35 ± 0.49	4.248	0.015*
Ratio2	0.31 ± 0.17	0.31 ± 0.23	0.49 ± 0.28	21.522	0.000**

*p < 0.05, **p < 0.01 1=primary compression trabecula, 2=overlapping area, 3=primary tension trabecula, 4=secondary compression trabecula, 5=the greater trochanter, 6=lateral of primary tension trabecula.

**Table 3:** Multiple comparison between 61–70 years old normal group and patients with and without diabetic fracture.

Area	(I) patient type	(J) patient type	(I) mean	(J) mean	D-value (I-J)	*p*	Cohen’s *d*值
1	Fracture with diabetes	Fracture without diabetes	360.610	369.775	-9.166	1.000	-0.087
	Fracture with diabetes	Normal	360.610	304.708	55.901	0.001**	0.532
	Fracture without diabetes	Normal	369.775	304.708	65.067	0.000**	0.620
2	Fracture with diabetes	Fracture without diabetes	308.920	292.874	16.046	0.501	0.195
	Fracture with diabetes	Normal	308.920	243.209	65.711	0.000**	0.799
	Fracture without diabetes	Normal	292.874	243.209	49.665	0.000**	0.604
3	Fracture with diabetes	Fracture without diabetes	93.568	86.350	7.219	1.000	0.136
	Fracture with diabetes	Normal	93.568	109.161	-15.593	0.112	-0.294
	Fracture without diabetes	Normal	86.350	109.161	-22.811	0.005**	-0.430
4	Fracture with diabetes	Fracture without diabetes	77.514	79.252	-1.738	1.000	-0.031
	Fracture with diabetes	Normal	77.514	101.485	-23.972	0.008**	-0.424
	Fracture without diabetes	Normal	79.252	101.485	-22.233	0.012*	-0.393
Ratio1	Fracture with diabetes	Fracture without diabetes	1.186	1.300	-0.114	0.138	-0.282
	Fracture with diabetes	Normal	1.186	1.349	-0.162	0.013*	-0.402
	Fracture without diabetes	Normal	1.300	1.349	-0.049	1.000	-0.120
Ratio2	Fracture with diabetes	Fracture without diabetes	0.310	0.310	-0.000	1.000	-0.001
	Fracture with diabetes	Normal	0.310	0.493	-0.184	0.000**	-0.777
	Fracture without diabetes	Normal	0.310	0.493	-0.184	0.000**	-0.776

*p < 0.05, **p < 0.01 1=primary compression trabecula, 2=overlapping area, 3=primary tension trabecula, 4=secondary compression trabecula, 5=the greater trochanter, 6=lateral of primary tension trabecula.

In **Table 2**, except for the greater trochanter (F = 2.71, p=0.07) and the lateral aspect of the primary tension trabeculae (F = 0.91, p=0.40), CT values in other regions differed significantly between groups (P<0.01). Fracture patients showed increased CT values in the primary compression trabecula (F = 12.16, p < 0.01) and overlapping area (F = 18.14, p < 0.01), as well as decreased CT values in the primary tension trabecula (F = 5.27, p < 0.01) and secondary compression trabecula (F = 5.94, p < 0.01). Ratio1 was also decreased (F = 21.52, p < 0.01). Specific changes are shown in **Figure 5**.

**Figure 5:**
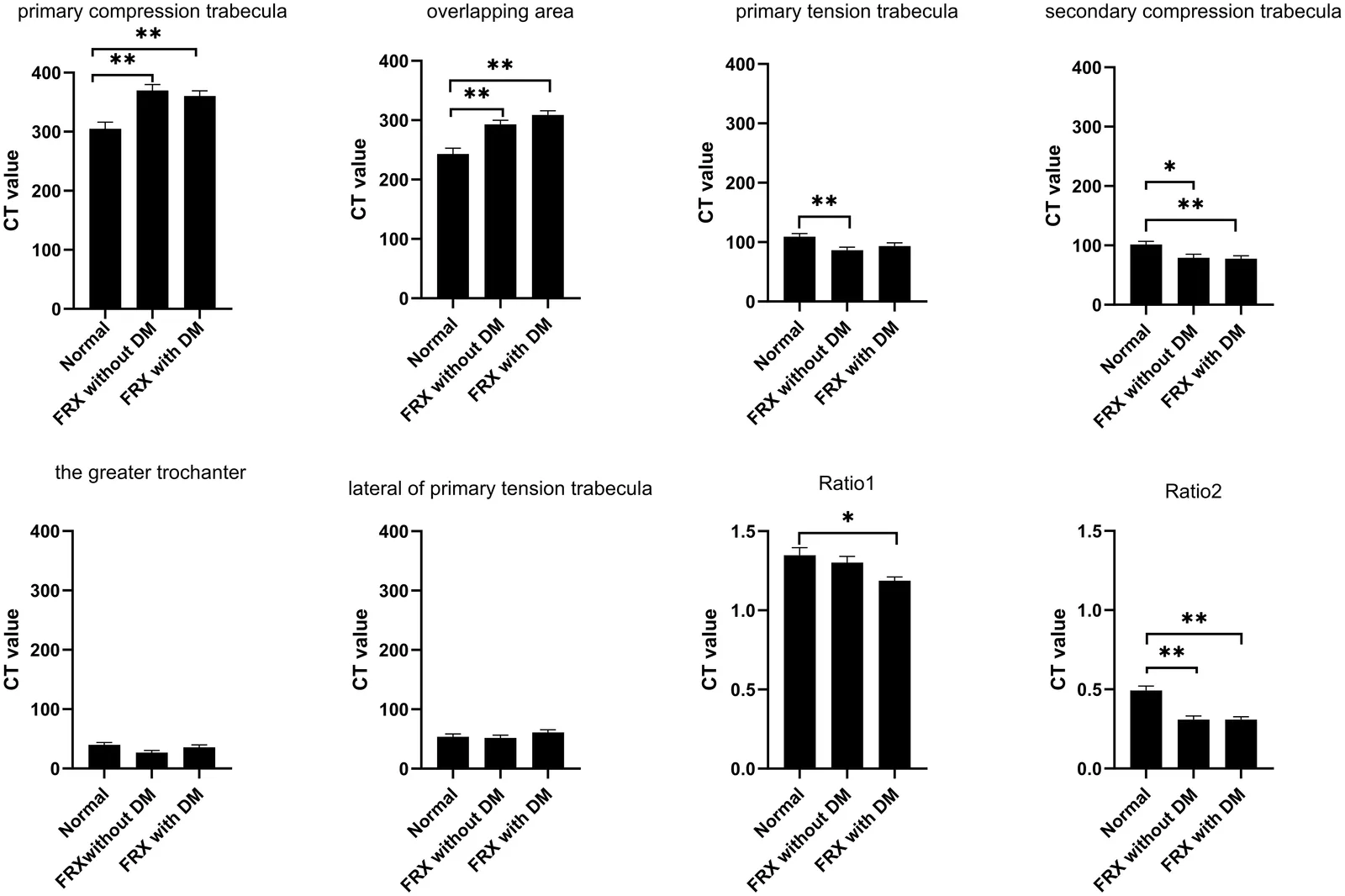
Changes of CT value of trabecular bone measurement areas in normal group and patients with and without diabetes fracture. FRX, Fracture; DM, Diabetes. (*P<0.05, **P<0.01, ***P<0.001).

Multiple comparisons between normal group (61–70 years) and fracture patients with and without diabetic showed no significant differences in CT values in any regions between two fracture groups (p>0.05) and the effect size was very small (**Table 3**). Ratio2 differed significantly between the normal group and both fracture groups (p < 0.01). For indices with statistically significant differences, Cohen’s d values ranged from 0.4 to 0.8, indicating moderate to large effect sizes, which suggests that the observed between-group differences are likely to be of practical significance.

### Interaction between sex and diabetes (two-way ANOVA)

To further investigate whether the effect of diabetes on trabecular parameters differs between sexes, a two-way ANOVA was performed with patient type (normal, non-diabetic fracture, diabetic fracture) and sex (male, female) as independent factors. As shown in **Tables 4** and **5**, a significant patient type × sex interaction was observed for CT values in the primary compression trabecular region (area 1) (F = 3.55, p < 0.05).

**Table 4:** Two-way analysis of variance (ANOVA).

Area	Patient type F	Patient type P	Sex F	Sex P	F (Patient type×sex interaction)	P (Patient type×sex interaction)
1	8.90	0.00**	0.19	0.67	3.55	0.03*
2	13.32	0.00**	2.51	0.11	2.14	0.12
3	6.38	0.00**	0.36	0.55	1.50	0.23
4	5.40	0.00**	6.42	0.01*	0.02	0.98
5	2.74	0.07	7.66	0.01**	0.72	0.49
6	0.71	0.49	2.52	0.11	0.55	0.58
Ratio1	4.03	0.02*	1.49	0.22	0.81	0.44
Ratio2	22.44	0.00**	1.50	0.22	1.59	0.20

*P<0.05, **P<0.01 1=primary compression trabecula, 2=overlapping area, 3=primary tension trabecula, 4=secondary compression trabecula, 5=the greater trochanter, 6=lateral of primary tension trabecula.

**Table 5:** Using sex as the grouping variable, test the simple effect of patient type at each level of sex.

Simple effect (patient type & sex)						
Patient type	Sex	Mean difference	Standard error (SE)	t	p	Cohen’s d
Fracture with diabetes	F - M	47.502	23.782	1.997	0.047	0.455
Fracture without diabetes	F - M	4.726	20.367	0.232	0.817	0.045
Normal	F - M	-36.094	20.572	-1.754	0.080	-0.346

Simple effects analysis revealed that among patients with diabetic fractures, females had significantly higher CT values in the primary compression trabecular region than males (t = 1.997, p < 0.05), with a medium effect size (Cohen’s d = 0.455). Using sex as the grouping variable, Bonferroni-corrected multiple comparisons were performed among the three groups (normal, non-diabetic fracture, diabetic fracture) within each sex. The results showed that, in both male and female patients, the differences between the diabetic fracture group and the non-diabetic fracture group were not statistically significant (p > 0.05) (**Figure 6**).

**Figure 6:**
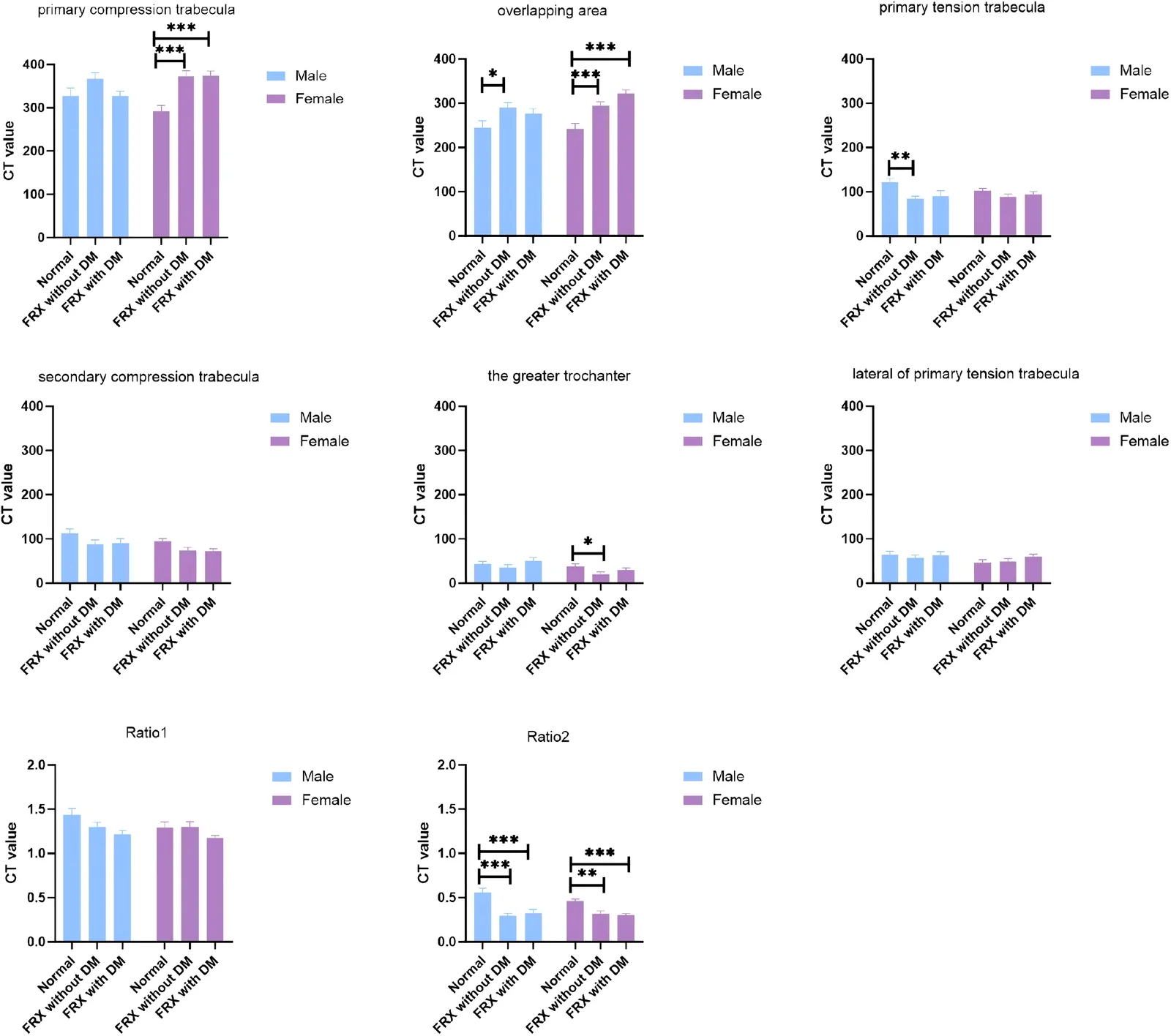
Changes of CT value of trabecular bone measurement areas in normal group and patients with or without diabetes fracture according to gender classification. (*P<0.05, **P<0.01, ***P<0.001).

These findings suggest that although a significant sex-by-diabetes interaction exists for the primary compression trabecula, the direct comparison between diabetic and non-diabetic fracture patients within the same sex did not reach statistical significance, indicating that the interaction may be driven by sex-specific baseline differences rather than a differential effect of diabetes per se.

### Comparison between femoral neck and intertrochanteric fractures

To further characterize the relationship between fracture type and trabecular redistribution, we compared CT values and ratio indices among the normal group (61–70 years, n=110), patients with intertrochanteric fractures (n=53), and patients with femoral neck fractures (n=150) (**Tables 6**, **7**).

**Table 6:** Multiple comparison between 61–70 years old normal group and patients with femoral neck and intertrochanteric fractures.

Area	Fracture area (mean ± standard deviation)			F	p
	Normal (n=110)	intertrochanteric (n=53)	femoral neck (n=150)		
1	304.71 ± 120.03	387.52 ± 100.96	357.76 ± 92.98	13.659	0.000**
2	243.21 ± 101.00	308.93 ± 74.50	297.26 ± 68.82	17.508	0.000**
3	109.16 ± 54.46	101.39 ± 66.66	85.56 ± 45.71	6.599	0.002**
4	101.49 ± 56.07	68.28 ± 70.90	82.04 ± 50.54	7.128	0.001**
5	39.72 ± 44.84	14.13 ± 31.48	36.70 ± 41.85	7.469	0.001**
6	53.45 ± 51.83	53.37 ± 48.99	56.86 ± 47.87	0.188	0.829
Ratio1	1.35 ± 0.49	1.31 ± 0.48	1.23 ± 0.29	3.039	0.049*
Ratio2	0.49 ± 0.28	0.34 ± 0.28	0.30 ± 0.17	22.312	0.000**

*p<0.05, **p<0.01 1=primary compression trabecula, 2=overlapping area, 3=primary tension trabecula, 4=secondary compression trabecula, 5=the greater trochanter, 6=lateral of primary tension trabecula.

**Table 7:** Multiple comparison between 61–70 years old normal group and patients with femoral neck and intertrochanteric fractures.

Area	(I) patient type	(J) patient type	(I) mean	(J) mean	D-value (I-J)	p	Cohen’s d
1	Normal	intertrochanteric	304.708	387.525	-82.816	0.000**	-0.792
	Normal	femoral neck	304.708	357.760	-53.052	0.000**	-0.507
	intertrochanteric	femoral neck	387.525	357.760	29.765	0.227	0.285
2	Normal	intertrochanteric	243.209	308.928	-65.719	0.000**	-0.797
	Normal	femoral neck	243.209	297.257	-54.048	0.000**	-0.656
	intertrochanteric	femoral neck	308.928	297.257	11.671	1.000	0.142
3	Normal	intertrochanteric	109.161	101.391	7.770	1.000	0.147
	Normal	femoral neck	109.161	85.559	23.602	0.001**	0.447
	intertrochanteric	femoral neck	101.391	85.559	15.832	0.185	0.300
4	Normal	intertrochanteric	101.485	68.281	33.204	0.001**	0.589
	Normal	femoral neck	101.485	82.039	19.446	0.019*	0.345
	intertrochanteric	femoral neck	68.281	82.039	-13.758	0.383	-0.244
5	Normal	intertrochanteric	39.718	14.126	25.592	0.001**	0.618
	Normal	femoral neck	39.718	36.699	3.019	1.000	0.073
	intertrochanteric	femoral neck	14.126	36.699	-22.573	0.002**	-0.545
6	Normal	intertrochanteric	53.453	53.368	0.085	1.000	0.002
	Normal	femoral neck	53.453	56.858	-3.405	1.000	-0.069
	intertrochanteric	femoral neck	53.368	56.858	-3.490	1.000	-0.071
Ratio1	Normal	intertrochanteric	1.349	1.308	0.040	1.000	0.099
	Normal	femoral neck	1.349	1.226	0.123	0.049*	0.303
	intertrochanteric	femoral neck	1.308	1.226	0.083	0.610	0.204
Ratio2	Normal	intertrochanteric	0.493	0.342	0.151	0.000**	0.639
	Normal	femoral neck	0.493	0.298	0.195	0.000**	0.828
	intertrochanteric	femoral neck	0.342	0.298	0.044	0.719	0.188

*p<0.05, **p<0.01 1=primary compression trabecula, 2=overlapping area, 3=primary tension trabecula, 4=secondary compression trabecula, 5=the greater trochanter, 6=lateral of primary tension trabecula.

As shown in **Table 6**, significant differences among the three groups were observed in most regions. Specifically, CT values of the primary compression trabecula (area 1) and overlapping area (area 2) were significantly higher in both fracture groups compared to the normal group, whereas CT values of the primary tension trabecula (area 3) and secondary compression trabecula (area 4) were lower in fracture patients (all p < 0.01). The greater trochanter (area 5) showed a marked decrease only in the intertrochanteric fracture group compared to normal controls (p < 0.01), while no significant difference was observed between the femoral neck fracture group and normal controls (p = 1.00). No significant differences were found among the three groups for the lateral aspect of the primary tension trabecula (area 6, p = 0.829).

Regarding the ratio indices, ratio1 showed a significant difference across groups (p = 0.049), with *post-hoc* analysis revealing a significant decrease in the femoral neck fracture group compared to the normal group (d = 0.303, p = 0.049), whereas the intertrochanteric fracture group did not differ significantly from either the normal group (p = 1.00) or the femoral neck group (p = 0.610). Ratio2 differed significantly among groups (p < 0.001), with both fracture groups showing markedly lower ratio2 values than the normal group (intertrochanteric: d = 0.639, p < 0.001; femoral neck: d = 0.828, p < 0.001), but no significant difference between the two fracture types (p = 0.719).

These findings suggest that while both fracture types are associated with trabecular redistribution, the magnitude and pattern of changes differ between intertrochanteric and femoral neck fractures.

## Discussion

Osteoporotic fractures of the proximal femur have serious consequences for patients, with one-year mortality around 25%. Increased mortality is associated with elderly populations (10). In clinical work, we observed that the distribution of trabecular bone in the proximal femur changes with age. By measuring HU values in different trabecular distribution areas, we aimed to infer the pattern of age-related trabecular changes in the proximal femur. Some scholars have analyzed the causes of hip joint fracture in the elderly through HU value changes, finding that HU values vary across pelvic regions and are strongly related to load distribution. These HU distribution patterns and age-related differences may explain the characteristic causes and patterns of acetabular fractures in the elderly and may aid surgical treatment (11).

### Effect of age on trabecular bone

During adolescence, trabeculae form a dense and regular mesh-like structure. The number of trabeculae is relatively stable and abundant, bone growth is rapid, and bone mass accumulation peaks, providing strong support and stability for rapid growth and development. With increasing age into adulthood, trabecular density and connectivity gradually decline, and the number slowly decreases, mainly due to bone resorption exceeding bone formation during remodeling. In middle and old age, this decline accelerates: trabecular number decreases significantly, the structure becomes sparse, connectivity weakens, bone fragility increases and fracture risk rises (12). In our study, although both types of trabecular bone decreased with age, the decrease in tension trabeculae was more severe, which may explain why elderly people are prone to fractures. This pattern aligns with the Singh index, in which “compressive trabeculae are preferentially preserved while tensile trabeculae are gradually lost, “ indicating that CT-based regional measurements capture selective trabecular loss (2). In our study, CT values in all trabecular regions decreased significantly in females after age 50 but remained relatively stable in males. The proposed mechanism is as follows. In females, bone mineral density peaks between 21 and 30 years of age. After menopause, estrogen deficiency shifts the balance toward bone resorption exceeding formation (13). This imbalance accelerates remodeling, increases mineralization heterogeneity, and induces focal trabecular thinning or thickening, ultimately leading to greater bone fragility and reduced strength (14–17).

### Sex differences in trabecular redistribution

In our study, ratio1 (primary compression trabecula/overlapping area) was not significantly affected by age in the overall cohort and remained relatively stable. Ratio1 remained stable in women, whereas it was higher in elderly men than in middle-aged and younger men. This sex difference can be explained by the distinct age-related trajectories of compression and tension trabeculae between males and females. Specifically, primary compression trabecular CT values decreased significantly in women after the age of 60 but remained relatively stable in men. Primary tension trabecular CT values gradually declined with age in women, whereas they remained relatively constant in men after the age of 40. Consequently, ratio1 differs between men and women. These findings are consistent with a study on elderly vertebral specimens, which reported that men had wider trabeculae and a higher bone area-to-tissue area (BA/TA, %) than women (18).

### Trabecular changes and osteoporotic fracture risk in the elderly

The present study found that although trabecular bone generally exhibits a declining trajectory with age, the relative content of compression trabeculae remains comparatively stable, possibly owing to gravitational loading. This observation aligns with previous findings from iliac bone specimens, which demonstrated that although trabecular bone area and the mean number of trabeculae per millimeter decline with age, trabecular anisotropy increases (19). The mechanical behavior of cancellous bone depends on both local inhomogeneities of tissue microarchitecture and trabecular anisotropy (20). With advancing age, increased trabecular anisotropy reflects a redistribution of trabecular bone, characterized by a relatively stable proportion of compression-bearing trabeculae and a progressive reduction in tension-bearing trabeculae.

Under physiological conditions, trabecular bone forms a porous network that enhances bone strength through optimized spatial arrangement and interconnectivity. However, with advancing age, trabecular bone density gradually decreases, the structure transitions from dense and homogeneous to sparse, and the architecture shifts from a radiating to a disordered pattern. These changes impair the ability of bone to withstand external forces, thereby increasing fracture risk. During aging, the preferential loss of transversely oriented trabeculae leads to a greater reduction in trabecular fatigue life than in stiffness or strength. This phenomenon may explain the long-standing but poorly characterized relationship between trabecular microarchitecture and clinical fractures (21). To compensate for bone loss, the body increases overall bone size, counteracting the effects of bone resorption at mechanically loaded sites (e.g., the medial cortex of the femoral neck) and protecting weight-bearing trabecular structures (e.g., primary compression trabeculae); this adaptive process is activated to maintain whole-bone stiffness under physiological loading (22).

### Effects of diabetes on trabecular redistribution

Diabetes accounts for approximately 10% of hip fracture cases and is associated with a 1.6- to 1.7-fold increased fracture risk. Notably, even when areal bone mineral density (aBMD) levels are high, osteoporotic fractures of the spine and hip still occur in diabetic patients (14). Diabetes and prior fragility fracture together confer the highest risk of death in the general population (23).

Our study focused on compression and tension trabeculae in the proximal femur of patients with diabetic osteoporosis versus senile osteoporosis, examining differences in trabecular redistribution between these two conditions. The results showed that diabetic patients lose the stability of ratio1, which declines significantly—indicating an inability to maintain relatively stable compression trabeculae—and this may explain their higher fracture risk.

Trabecular bone microarchitecture consists of a single trabecular network that can be classified into plate-like and rod-like structures (24). Plate-like trabeculae support most of the axial load during trabecular failure (25), whereas rod-like trabeculae are primarily oriented laterally and contribute less to stiffness and strength. Individual trabecular segmentation (ITS) studies have demonstrated that in osteoporosis and other metabolic bone diseases, trabecular microstructure transitions from plate-like to rod-like, suggesting that this morphological change is an important contributor to age- and osteoporosis-related bone fragility (26). In cancellous bone from patients with type 2 diabetes mellitus (T2DM), micro-damage is more evenly distributed between plates and rods, whereas in non-diabetic specimens, rods accumulate more damage (24). Accumulation of advanced glycation end products (AGEs) in diabetic patients may underlie the deterioration of cortical geometry and trabecular microstructure (27). Collagen cross-linking occurs via two mechanisms: enzymatic and non-enzymatic. Non-enzymatic cross-linking results from the production of AGEs (28). Compared with non-diabetic samples, T2DM samples exhibit higher pentosidine concentrations, higher sugar-to-matrix ratios, and a higher ratio of non-enzymatic cross-linking to mature enzymatic cross-linking, all of which render T2DM bone more brittle and less resistant to fracture (29). Total damage accumulation did not differ between the two groups, which may be attributable to a mitigating effect of glycosylation on post-yield micro-damage formation (24). This may explain why the reduction of trabecular bone in diabetic patients is generalized, with no obvious redistribution phenomenon observed in the present study. It is worth noting, however, that micro-trabecular analysis cannot easily capture the influence of macroscopic gravity or tension on trabecular arrangement—a gap that our study aimed to address.

### Fracture type-specific trabecular redistribution patterns

Our subgroup analysis comparing intertrochanteric and femoral neck fractures revealed interesting differences in trabecular redistribution patterns. Notably, the greater trochanter (area 5) showed a marked decrease in CT values only in patients with intertrochanteric fractures, but not in those with femoral neck fractures, compared to normal controls. This finding is biomechanically plausible, as the greater trochanter serves as the insertion site for the hip abductors (gluteus medius and minimus), and intertrochanteric fractures typically involve the region from the greater trochanter to the lesser trochanter (30, 31). The selective reduction of trabecular density at the greater trochanter in intertrochanteric fractures may reflect localized bone loss that predisposes to this specific fracture type.

Conversely, patients with femoral neck fractures exhibited a more pronounced reduction in ratio1 (primary compression trabecula/overlapping area) compared to normal controls (d = 0.303, p = 0.049), whereas ratio1 in intertrochanteric fracture patients did not differ significantly from controls. This suggests that the relative stability of compression trabeculae is preferentially compromised in femoral neck fractures, which may be related to the distinct biomechanical environment of the femoral neck region under weight-bearing and shear stresses (32).

Both fracture types demonstrated similarly significant reductions in ratio2 (primary tension trabecula/overlapping area), consistent with our overall finding that age-related tension trabecular loss is a common feature in elderly fracture patients regardless of fracture location. These fracture type-specific differences, while exploratory, highlight the potential value of region-specific trabecular assessment in understanding the pathogenesis of different hip fracture patterns.Recent work by Kaya and Karabak (2026) demonstrated that morphometric parameters and bone quality are closely associated with elderly hip fracture patterns, further supporting the importance of structural bone characteristics in fracture-type analysis (33). Larger prospective studies are warranted to confirm these observations and to determine whether trabecular parameters can predict fracture type.

### Limitations

A *post-hoc* power calculation for the one-way ANOVA of ratio1 revealed that the statistical power was 62% at α=0.05, indicating that the study may have been insufficiently powered to detect small-to-moderate age-related changes in compression trabecular ratio. Therefore, the non-significant finding (F = 1.84, p=0.121) should be interpreted cautiously and does not constitute evidence of stability. Future studies with larger sample sizes are warranted to confirm this observation.

Several limitations should be acknowledged. First, this was a retrospective, single-center study, which may limit generalizability. Second, the HU-based ratio indices were not validated against DXA, QCT, or micro-CT. Third, no adjustment was made for potential confounders such as medication history, physical activity, or lifestyle factors. Fourth, the cross-sectional design precludes causal inference; findings are associative only. Fifth, the exclusively Chinese cohort limits generalizability to other ethnic groups. Sixth, the use of 2D clinical CT images cannot capture true 3D trabecular microarchitecture (e.g., trabecular number, connectivity, plate-to-rod transition). Finally, detailed data on menopausal status, individual BMI values, and diabetes characteristics (type, duration, glycemic control) were unavailable. Future prospective, multi-center studies with 3D imaging and comprehensive clinical data are needed to validate our findings.

## Data Availability

The raw data supporting the conclusions of this article will be made available by the authors, without undue reservation.

## Supplementary material

Supplementary Table 1 Intraclass correlation coefficient for CT values of trabecular bone in each region measured by two observers. 1=primary compression trabecula, 2=overlapping area, 3=primary tension trabecula, 4=secondary compression trabecula, 5=the greater trochanter, 6=lateral of primary tension trabecula.

Supplementary Table 2 Intraclass correlation coefficient for CT values of trabecular bone in each region measured by the same observer at different times. 1=primary compression trabecula, 2=overlapping area, 3=primary tension trabecula, 4=secondary compression trabecula, 5=the greater trochanter, 6=lateral of primary tension trabecula.
