# Supplementary material for: CT-based age-related trabecular redistribution in the proximal femur and its association with fracture patterns in elderly individuals with and without diabetes
Source: Front Endocrinol (Lausanne). 2026 Sep 29;17:1830903. doi: 10.3389/fendo.2026.1830903 (PMC13662763; doi:10.3389/fendo.2026.1830903)
Supplement: Supplementary file 1 [file Table1.docx]

Table S1: Intraclass correlation coefficient for CT values of trabecular bone in each region measured by two observers

| Area | Intraclass correlation coefficient  (Single Measures) | 95% confidence interval  (Single Measures) | Intraclass correlation coefficient  (Average Measures) | 95% confidence interval  (Average Measures) | P |
| --- | --- | --- | --- | --- | --- |
| 1 | 0.841 | 0.647 ~ 0.934 | 0.914 | 0.786 ~ 0.966 | ＜0.001 |
| 2 | 0.878 | 0.719 ~ 0.950 | 0.935 | 0.836 ~ 0.974 | ＜0.001 |
| 3 | 0.941 | 0.858 ~ 0.976 | 0.969 | 0.923 ~ 0.988 | ＜0.001 |
| 4 | 0.910 | 0.791 ~ 0.963 | 0.953 | 0.883 ~ 0.981 | ＜0.001 |
| 5 | 0.933 | 0.838 ~ 0.973 | 0.965 | 0.912 ~ 0.986 | ＜0.001 |
| 6 | 0.911 | 0.791 ~ 0.964 | 0.953 | 0.883 ~ 0.982 | ＜0.001 |
| Ratio1 | 0.820 | 0.606 ~ 0.924 | 0.901 | 0.755 ~ 0.961 | ＜0.001 |
| Ratio2 | 0.829 | 0.624 ~ 0.928 | 0.907 | 0.768 ~ 0.963 | ＜0.001 |

1=primary compression trabecula, 2=overlapping area, 3=primary tension trabecula, 4=secondary compression trabecula, 5=the greater trochanter ，6=lateral of primary tension trabecula.
