# Supplementary material for: CT-based age-related trabecular redistribution in the proximal femur and its association with fracture patterns in elderly individuals with and without diabetes
Source: Front Endocrinol (Lausanne). 2026 Sep 29;17:1830903. doi: 10.3389/fendo.2026.1830903 (PMC13662763; doi:10.3389/fendo.2026.1830903)
Supplement: Supplementary file 2 [file Table2.docx]

Table S2: Intraclass correlation coefficient for CT values of trabecular bone in each region measured by the same observer at different times

| Area | Intraclass correlation coefficient  (Single Measures) | 95% confidence interval  (Single Measures) | Intraclass correlation coefficient  (Average Measures) | 95% confidence interval  (Average Measures) | P |
| --- | --- | --- | --- | --- | --- |
| 1 | 0.860 | 0.679 ~ 0.942 | 0.925 | 0.809 ~ 0.970 | ＜0.001 |
| 2 | 0.938 | 0.851 ~ 0.975 | 0.968 | 0.919 ~ 0.987 | ＜0.001 |
| 3 | 0.904 | 0.774 ~ 0.961 | 0.950 | 0.873 ~ 0.980 | ＜0.001 |
| 4 | 0.906 | 0.762 ~ 0.963 | 0.951 | 0.865 ~ 0.981 | ＜0.001 |
| 5 | 0.846 | 0.653 ~ 0.936 | 0.917 | 0.790 ~ 0.967 | ＜0.001 |
| 6 | 0.819 | 0.602 ~ 0.924 | 0.901 | 0.752 ~ 0.960 | ＜0.001 |
| Ratio1 | 0.800 | 0.558 ~ 0.916 | 0.889 | 0.717 ~ 0.956 | ＜0.001 |
| Ratio2 | 0.839 | 0.641 ~ 0.933 | 0.913 | 0.781 ~ 0.965 | ＜0.001 |

1=primary compression trabecula, 2=overlapping area, 3=primary tension trabecula, 4=secondary compression trabecula, 5=the greater trochanter ，6=lateral of primary tension trabecula.
